# Patients’ experiences of referral for colorectal cancer

**DOI:** 10.1186/1471-2296-14-124

**Published:** 2013-08-26

**Authors:** Shane W Pascoe, Craig Veitch, Lisa J Crossland, Justin J Beilby, Allan Spigelman, John Stubbs, Mark F Harris

**Affiliations:** 1 UNSW Research Centre for Primary Health Care and Equity, School of Public Health and Community Medicine, UNSW, Sydney, Australia; 2 Faculty of Health Sciences, The University of Sydney, Sydney, Australia; 3 Department of Rural Health, Mount Isa Centre for Rural and Remote Health, James Cook University, Mount Isa, Qld, Australia; 4 Faculty of Health Sciences, University of Adelaide, Adelaide, Australia; 5 St Vincent’s & Mater Health, UNSW, St Vincent’s Clinical School, Sydney, NSW, Australia; 6 CanSpeak, Sydney, Australia

**Keywords:** Colorectal neoplasm, Referral and consultation, Patients, Qualitative research

## Abstract

**Background:**

Outcomes for colorectal cancer patients vary significantly. Compared to other countries, Australia has a good record with patient outcomes, yet there is little information available on the referral pathway. This paper explores the views of Australian patients and their experiences of referral for colorectal cancer treatment following diagnosis; the aim was to improve our understanding of the referral pathway and guide the development of future interventions.

**Methods:**

A purposive sampling strategy was used, recruiting 29 patients representing urban and rural areas from 3 Australian states who participated in 4 focus groups. Seven patients provided individual interviews to supplement the data. Recordings were transcribed verbatim, data was coded with NVivo software and analysed thematically before deductive analysis.

**Results:**

Four aspects of the referral process were identified by patients, namely detection/diagnosis, referral for initial treatment/specialist care, the roles of the GP/specialist, and the patient’s perceived involvement in the process. The referral process was characterised by a lack of patient involvement, with few examples of shared decision-making and few examples of limited choice. However, patients did not always feel they had the knowledge to make informed decisions. Information exchange was highly valued by patients when it occurred, and it increased their satisfaction with the process. Other factors mediating care included the use of the public versus private health system, the quality of information exchange (GP to specialist and GP to patient), continuity of care between GP and specialist, and the extent of information provision when patients moved between specialist and GP care.

**Conclusions:**

Patients described poor GP continuity, ad hoc organisational systems and limited information exchange, at both interpersonal and inter-organisational levels, all leading to sub-optimal care. Implementation of a system of information feedback to GPs and engagement with them might improve information exchange for patients, enabling them to be more involved in improved referral outcomes.

## Background

Colorectal cancer (CRC) is a more commonly occurring cancer in Australia and represents a significant and growing health burden [1-4]. Effective communication across the primary/secondary interface is vital for the planning and delivery of appropriate cancer patient care [5]. Even within secondary care, there is considerable variation in the implementation of effective multi-disciplinary care [6]. Primary care continues its important role since the Australian Government commenced its National Bowel Cancer Screening Initiative in 2006, where those turning 55 or 65 are screened using a faecal occult blood tests (FOBT). People with positive results are sent to their GP, who refers them for sigmoidoscopy [7]. For CRC patients, satisfaction is inversely related to both diagnostic and treatment delay and delay in either area may result in psychological distress [8] and poorer outcomes [5].

Being informed of the diagnosis is, in itself distressing and although healthcare systems in Australia do well in comparison to other countries in terms of survival [6], improvements in the processes of care are needed. Australia has a universal health care system funded at a state and federal level providing access and choice through private sector involvement in treatment. The national health insurance scheme, “Medicare”, funds GP care and treatment in public hospitals. About half of Australians also have private insurance which funds cancer treatment in private hospitals. (5) GPs refer patients with symptoms for a diagnostic colonoscopy and following diagnosis continue to be involved in the management of patients undergoing cancer treatment. [7,8].

Referral is the process of directing a patient to access care, informing the carer, and assuming an on-going role in that care. Medical referrals for CRC in Australia are most frequently made to surgeons, followed by gastroenterologists and oncologists [9] and while GPs in the UK have a significant role in determining to which specialist a patient is referred, patients can travel multiple pathways prior and subsequent to diagnosis [10]. As these referral pathways can span primary and secondary care and public and private providers, patients often move back and forth between services. Good co-ordination between all parties is therefore a priority [11,12].

CRC patients report poor co-ordination between primary and secondary care and consider that this deficiency compromises the referral process [13]. A patient’s level of education, socio-economic status, gender, cultural background, and access to services can also influence the length of time before treatment commences [14,15]. Continuity also has an effect on diagnostic delay: individuals with a regular healthcare provider are more likely to seek medical attention earlier than those who do not [15]. These variables are important in any analysis of the CRC referral pathway.

Patients typically value rapid diagnosis [16], and policies both in Australia and elsewhere aim to improve outcomes by placing patients and service users at the centre of decisions about their care [17]. Screening programs such as the Australian National CRC Screening Program seek to make the diagnosis of CRC earlier [18]. In the UK, only a minority of patients are referred using the optimal pathway [19-21]. In Australia, information regarding the quality or timeliness of referral pathways is limited.

This study aimed to explore Australian patients’ perspectives of the referral pathway when they first receive the diagnosis of CRC, and to describe their expectations regarding referral to specialist services in order to improve the patient pathway.

## Methods

The study used a conceptual framework of the care pathway from diagnosis to follow-up [22] to guide the approach taken with the data. The approach was informed by a constructivist perspective, considering the experiences shared between researchers and participants as a priority.

### Sample

Purposive sampling was used to ensure a diverse range of views. The purposive sampling technique involved contacting health care professionals and patient advocates in existing health care organisations. Purposive sampling was used to ensure inclusion of a range of patients by age, gender, geography (urban/rural and by state), and type of health care (public/private). Patients were recruited from support groups and by hospital staff, in person and via telephone, in both public and private hospitals in 3 Australian states (New South Wales (NSW), South Australia (SA) and Queensland (Qld)). No data was available on those who chose not to participate. The University of New South Wales (UNSW) Human Research Ethics Committee approved the study (HC08065).

### Data collection

Focus groups were conducted to encourage participants to discuss with their peers factors influencing their referral [23]. It was anticipated that collectively, participants would have a range of experiences of referral. The focus groups and individual interviews used a series of questions and prompts designed to take patients through their experience from the time they developed their first symptoms to the current day (see Appendix A for the focus group questions). Patients were given the choice of attending a group or an individual interview (face-to-face or via telephone), to take into account patient preferences for disclosure and limitations due to access or mobility. Consent was obtained to record both focus group discussions and interviews; these were transcribed by a service with experience in this area, and all were checked by the lead author. The focus groups were conducted sequentially over a 14-month period from June 2009 to August 2010. The interviewers were all health services researchers experienced in qualitative methods. Two of the interviewers (SP and JB) had clinical treatment experience and had very good communication skills. Data saturation was achieved at the conclusion of the fourth focus group, where no new ideas were being added and the group reported similar experiences and highlighted similar themes to the preceding sample. It was noted that participants from public hospitals were under-represented and therefore additional individual interviews from these patients were included.

### Data analysis

Data was coded using NVivo software [24]. The analysis was thematic, based on a social constructionist epistemology [25]. After SP had read the transcripts, an agreed coding framework was developed in consultation with another author (MH).

Codes were reviewed for duplication and clarity and emergent themes were added to the coding framework to ensure completeness. The themes were then checked and verified, and differences in the accuracy or completeness of themes were resolved by discussion among the authors (LC, SP and MH). A constant comparison method was used to improve the internal consistency of codes. Other methods used to improve the reliability and validity of coding included a measure of word repetitions; further, the data were examined to identify missing information, metaphors, similes, and analogies. An assessment of connectors (e.g. ‘since’, ‘because’, ‘as’ ‘implies’, ‘means’, and ‘is one of’), and of unmarked text was also completed. A 20 % sample of the coding was cross-checked by a second researcher (MH). Themes were supported by direct quotes from patients in the focus groups and from individual interviews. Patients’ comments were identified with an individual number and as having public or private health insurance where appropriate.

## Results

Twenty-nine patients participated in four focus groups. Seven additional individual interviews supplemented the sample. In total there were 22 female and 14 male participants (see Table 1). In the South Australian focus group, 3 of the 4 patients were privately insured; in the NSW focus group all patients were privately insured; in the Qld focus groups 5 of the 7 participants were privately insured.

**Table 1:** Gender and number of focus group and individual interview participants

**State**	**Number of male patients**	**Number of female patients**	**Number of carers**	**Total number of patients**
Focus Groups				
Qld – Rural	7	4	3	11
Qld – Urban	1	8	0	9
NSW	2	2	1	4
SA	1	4	1	5
Individual Interviews				
Qld	0	1†	0	1
NSW	2	3‡	0	5
SA	1	0	0	1

^†^, 1 Public Patient; ^‡^, 1 of the 3 was a public patient with the remainder private.

### Referral pathway

Patients spoke about four aspects of the referral process, namely detection/diagnosis, referral for initial treatment/specialist care, the roles of the GP/specialist, and the patient’s perceived involvement in the process.

### Initial contact with GP at detection/diagnosis

Patients reported presenting to their GP following symptoms or bowel screening. Changes in the severity of symptoms pre-diagnosis often prompted patients to initiate contact with their GP. A general practice consultation was usually the first health service contact before a diagnosis, although some patients reported other avenues such as bowel screening services. Regardless of the initial point of contact, the GP was usually involved in referral at some stage.

“did the bowel screen and I came up positive and they said ‘contact your GP’ which I did and he sent me off to a proctologist for a colonoscopy” – NSW individual interview, male [private].

### Referral for initial treatment/specialist care

Patients reported that GPs’ used existing networks to identify a surgeon. It was not evident to participants that the target for referral was tailored to their specific needs. However, patients perceived that their feedback helped GPs in developing and maintaining these networks.

*“I just went to the one he always sends me to” – NSW, urban, individual interview, female 1 [private*].

“*I think the GPs sometimes go on feedback from patients*” – *NSW*, *urban group*, *female 1* [*private*].

Patients’ reported that easy access was important. They perceived that this, rather than other factors, strongly influenced GPs’ decisions as to where to refer.

“*He knows that me and my husband have a car* … *I think he usually chooses somebody reasonably local*.” – *NSW*, *urban individual interview*, *female 1* [*private*].

Gender issues might also influence accessing services for detection and could increase the need for a speedy referral. Men stated that services were better geared for female health issues. They also stated that actual need for treatment was under-recognised by men.

“*There is so much information pushed down about breast cancer* … *cervical cancer and so the ladies are more prepared to go and get advice or information*” – *NSW*, *urban group*, *male 5* [*private*].

“*I*’*ll get around to that one of these days* … *You know it*’*s the old saying you know*, *she*’*ll be right mate*, *it won*’*t happen to me*. – *NSW*, *individual*, *male* [*private*].

Between detection and diagnosis, patients described a range of waiting times. Private patients were often referred and saw a specialist quickly. Aspects of healthcare organisation (specifically the difference in accessing public and private systems) were considered by patients to be problematic. Patients in the public system usually had longer waiting times. Some patients considered it too costly to go outside the public system for referral.

“*Two or three days later I was with a specialist* … *and a week later I was in the hospital for the colonoscopy and a week later I was on the operating table* … *It was very fast*” – *NSW*, *group*, *female 1* [*Private*].

“*Reasonably urgent colonoscopy*, *that will be four weeks to do the colonoscopy*” – *SA*, *group*, *female 2* [*public*].

“‘*Would you have it done privately*?’ *and I said* ‘*No* … *it*’*d cost me a bomb*’.” – *Qld*, *rural group*, *female 4* [*public*].

Patients identified sources of dissatisfaction as poor continuity of care for themselves and individual GPs, especially where this was a cause of slower referral. Early and rapid referral was associated with greater patient satisfaction with their GP’s care.

“[*the patient waited*] *Two weeks to see a doctor so I went and pleaded my case and saw a* [*different*] *doctor* … *within a week*. *She wouldn*’*t even look at it*” – *Qld*, *rural group*, *male 7* [*private*].

### Specialist care/ professional roles

Communication between patient and surgeon and the quality of information provided were important in determining patient satisfaction with treatment. These factors also influenced how confidently patients made decisions about which treatment to accept. Poor communication was associated with poor satisfaction with treatment.

“*So at every step* [*e*.*g*. *at consultation with colonoscopist*] *it seemed the information about me had never got through*” – *SA*, *group*, *female 2* [*public*].

“*I suppose I worried about it* … *then I was explained all the statistics*[*by the surgeon*] – *you know*, *I was given a lot of information*.” – *NSW*, *urban group*, *male 4* [*private*].

Continuity and consistency of care were impaired by lack of communication and difficulty in accessing secondary care services. Perceived lack of communication between the specialist and GP hindered the GP’s ability to effect a referral.

“*I still haven*’*t got an appointment* [*e*.*g*. *a consultation with a colonoscopist* /*surgeon* ], *and at no time did it occur to me that I should have been ringing Royal Brisbane directly*. *I thought I had to go through her* [*the GP*]” – *Qld*, *urban group*, *female 8* [*private*].

Throughout the treatment process, patients felt that surgeons and GPs had distinct roles. Patients saw surgeons as conducting follow-up, answering questions, and providing information. Patients’ descriptions of the GP’s role varied, and included medication management, management of psychosocial issues, and provision of treatment advice.

“*I was always under the care of the oncologist afterwards* … *if it was not of an urgent nature he*’*d just say* ‘*go and see your GP*’, *you know*, *which is OK*.” – *NSW*, *urban group*, *male 4* [*public*].

Some patients directly contradicted each other in the range of services they were referred to, especially regarding allied health.

*Who were you referred to*? - *Interviewer.*

*Nobody* – *NSW*, *urban group*, *female 1* [*private*].

*Dietician*, *psychologist* -– *NSW*, *urban group*, *male 4* [*private*].

*Nobody else really* - – *NSW*, *urban group*, *male 6* [*private*].

### Patient involvement in the referral process

Patients stated that it was more often the GP who made the choice as to whom they were referred. Some patients felt that, although theoretically they had some level of choice (especially if they had private insurance), in reality, they had little involvement in decision-making. They lacked opportunities to contribute to the decision-making process. Importantly, patients felt that they lacked the information required to make an informed decision.

“*Had little choice of who I saw and where I went*. *GP made those decisions*. *We didn*’*t discuss it*.” – *Qld*, *individual interview*, *female* [*public*].

GPs rarely discussed the variables and considerations taken into account in making the decision regarding whom to refer to. The only example was from a rural patient:

“*He* [*GP*] *said*, ‘*Well there*’*s a chap in town but I don*’*t think* … *you know*, *I think it*’*s a bit above* [*the local surgeon*’*s experience*] *and you probably need more intensive care* … *somebody who simply specialises*’… *I*’*m sure he had to mention the local bloke*.” – *Qld*, *rural group*, *male 2* [*public*].

Positive experiences were associated with information exchange between GP and patient during treatment and planning the involvement of other members of a multidisciplinary team. Information exchange was a specific strategy identified by patients to improve care. Anticipatory and preparatory information was perceived to be crucial to effective care and good outcomes.

“*It*’*s lovely to be so included* … *when they had decided to be aggressive* … *the radiologist*, *the oncologist all these people* … *the stoma therapist* … *saying* ‘*Look*, *you*’*re going to have to do this*, *and you*’*re probably going to have a bag*’ … *anyhow I*’*m quite convinced that if I hadn*’*t have gone down that road* … *I*’*d be dead*” – *Qld*, *rural group*, *male 2* [*public*].

“*There needs to be multidiscipline routine care* … *where they actually have a team conference and talk about the patient as the*, *the centre*, *of the care*” – *SA*, *group*, *female 2* [*public*].

## Discussion

The referral pathway for CRC diagnosis and treatment is complex. Initial decisions on this pathway can make a difference to outcomes for patients in this study. The study explored CRC patients’ perspectives of the referral pathway during diagnosis, their expectations regarding referral to specialist services, their experiences of existing services, and the need for services. Patient awareness of a change in symptoms (patient interval) [26] was an influence on the diagnostic interval between symptoms, referral and diagnosis, as was the perceived quality of the initial contact with the GP. The existence of a long-term relationship between patient and GP and the availability of private health insurance were also important determinants of satisfaction with the referral process.

The Anderson Total Patient Delay Model developed by Walter identifies events, processes, intervals and other contributing factors as important influences on the diagnostic interval [27]. Our findings provide support for this model. Patients in this study detected change events (e.g. symptoms), which were described as a reason to discuss the symptom with the healthcare provider. The processes themselves were patient-initiated, but choice within the system was limited.

Patients often stated that they lacked an opportunity to contribute to the decision-making process, and lacked the information necessary to make well-informed decisions. This negatively influenced their treatment experience. Good communication between all parties was related to a feeling of patient satisfaction with the experience of referral, and to the ease with which patients made decisions about their treatment. It is possible to overcome barriers to access and gaps in quality, through the use of strategies such as engagement of community members and “peer navigators” within the health system, and active GP support of patients throughout the total interval, which was particularly useful for rural patients in this study and elsewhere [28,29].

The steps within the referral pathway are affected by contributing factors. The relationship with the GP was considered important by patients in this study, as has been reported in comparable studies, in that continuity and good communication skills influence timely diagnosis and the subsequent referral pathway [15].

Patients stated that during the diagnostic interval, healthcare provider and system factors such as better continuity of care with GPs and access to private health insurance facilitated ‘early’ and ‘appropriate’ referral. Patients who had been managed in the public system reported less satisfaction with the care provided, largely because of delays and poor communication. Waiting times in the public health system were an issue for most patients in this study. Patients reported that their GPs had been able to influence the speed of the referral (duration of primary care interval). However, GPs needed the capacity to overcome barriers within the public health system.

This study drew on patients’ experiences in three Australian states covering rural and urban areas across the public and private patient populations. The sample included patients attending public and private hospitals, support groups and consumer advocacy groups. The under-representation of males in the study is a reflection of the greater use of primary care by female patients and the reluctance of men to reflect on their experience with cancer. The results therefore reflect the views of patients who used these services. It is possible that such participants had atypical expectations and experiences. The study had little information from specific sub-groups in the Australian population such as those from Indigenous and culturally/linguistically diverse backgrounds. Although there was a high level of consistency and agreement amongst the patients regarding factors influencing the patient, doctor and system interval, the findings may not be generalisable to all patients.

## Conclusions

In summary, patients perceived that a poor quality relationship with their GP, poor continuity, limited information exchange both at interpersonal and at inter-organisational level, all contributed to sub-optimal care. Continuity and good information transfer were mediators of the perceived quality of the patient experience of referral, the period following diagnosis, and beyond. Referral from general practice is the main pathway to specialist services in Australia. Outcomes vary for patients however it is not known to what extent patients believe GP and organisational systems contribute to care. This paper describes poor GP continuity and limited information exchange all leading to sub-optimal care in the patients’ experience. Greater involvement through shared decision making, choice and information exchange all influence the perception of improved outcomes for patients.

Further research is needed to explore the continuity of involvement GPs with the care of patients across the cancer journey and their communication with medical specialists and specialist services. The sharing of audit information with both patients and GPs may help to ensure a more systematic approach within the treatment pathway. Quality improvement systems are needed in both public and private health services to ensure that patients experience a predictable and timely pathway to quality care.

The implication of these findings for GPs is that it is desirable to ensure continuity and engagement with their CRC patients who are navigating the broader cancer care system and beyond. Given outcomes vary according to the patients’ place of residence and clinician variables, the findings regarding the importance of the patients relationship with their GP has significant implications for care coordination and psychosocial care across the total interval [26]. This may improve outcomes for patients by decreasing delays and improving satisfaction, perception of choice and shared decision making. Patients also need more information about the referral service and surgeon to whom they are being referred. Unfortunately, such information is often unavailable to the GP in a form that might help them make decisions about to whom to refer to (e.g. surgeon volume) [30]. An integrated system bridging private providers, the local hospital, and primary health care networks could facilitate this information transfer and include general practice more effectively in an often distressing area of care.

### Ethics

Ethics approval was provided by the UNSW Human Research Ethics Committee.

## Appendix A

### Focus Group Questions

1. What was your relationship like with your GP before you were first diagnosed with CRC?

Prompts:

i. Assessment/diagnosis

ii. Communication

2. How was your diagnosis communicated?

Prompts:

i. Level of information/understanding

ii. Follow-up/next step

3. What do you think are the priorities for health care professionals in referring patients after they are newly diagnosed with CRC?

Prompts:

i. Communication

ii. Access (appointment times)

iii. Proximity

iv. Follow-up

4. What treatment options were discussed after diagnosis?

Prompts:

i. Expectations

ii. Effectiveness

iii. Accessibility

iv. Cost

5. How was the specialist to whom you were referred chosen?

Prompts:

i. What was your influence over the referral decision?

ii. Was your GP involved?

iii. Was there a directory of CRC specialists available?

6. How did the referring doctor communicate with the specialist service?

Prompts:

i. Letter

ii. Email

iii. Fax

iv. Phone

v. In person

7. What was your experience when you first attended the specialist appointment?

8. How was the communication between the specialist service and the GP?

Prompts:

i. When first seen

ii. During your treatment

iii. Follow-up

9. Did you see your GP while you were receiving specialist cancer treatment?

Prompts:

i. What for?

ii. What role did your GP have?

iii. What did you think about this?

10. What were your expectations after treatment?

Prompts:

i. Feedback

ii. Information

iii. Access

iv. Subsequent referral (Who was involved?)

11. How was the communication back to your GP and/or referring doctor?

Prompts:

i. Did your GP know about all your treatment/prescriptions?

ii. The next step

12. What were your experiences after treatment?

Prompts:

i. Communication to family/carer

ii. Symptom management

iii. Information

iv. Access

v. Subsequent referral

E.g. Support network

13. What are your preferences regarding referral, looking back?

Prompts:

i. Looking back, did your expectations match your experience?

ii. What methods would be best to meet your referral needs? (e.g. telehealth, Internet)

iii. What types of referral issues and barriers does your health area encounter (e.g. geographic barriers, rural issues, demographics)?

iv. Are there specific resources you feel you should have in your community for cancer care, but don’t currently have?

14. Final comments

* Anything that we’ve missed?

 – Partner/carer involvement

* Anything you would like to add?

## Abbreviations

CRC: Colorectal cancer; GP: General practitioner; NSW: New South Wales, Australia; SA: South Australia, Australia; Qld: Queensland, Australia; UNSW: University of New South Wales.

## Competing interests

The authors declare that they have no competing interests.

## Authors’ contributions

SP helped conceive the study, carried out the studies, participated in the focus groups and drafted the manuscript. MH helped conceive the study, assisted with the data analysis and drafted the manuscript. LC participated in the focus groups and helped to draft the manuscript. JB helped conceive the study, participated in the focus groups and helped to draft the manuscript. CV helped conceive the study, participated in its design and co-ordination, and helped to draft the manuscript. AS helped conceive the study, participated in its design and co-ordination, and helped to draft the manuscript. All authors read and approved the final manuscript.

## Authors’ information

Dianne L O’Connell: Cancer Research Division, Cancer Council NSW, David Goldsbury: Cancer Research Division, Cancer Council NSW, Michael B Barton: Collaboration for Cancer Outcomes Research & Evaluation, Liverpool Health Service, Liverpool, NSW, Ian Olver: Cancer Council Australia, NSW, David Weller: School of Clinical Sciences and Community Health, University of Edinburgh, Scotland.

## Pre-publication history

The pre-publication history for this paper can be accessed here:

http://www.biomedcentral.com/1471-2296/14/124/prepub
